# Assessment of platelet-rich fibrin in the maintenance and recovery of cell viability of the periodontal ligament

**DOI:** 10.1038/s41598-019-55930-0

**Published:** 2019-12-20

**Authors:** Lorena Bortolini Navarro, Fabiane Barchiki, Wilson Navarro Junior, Everdan Carneiro, Ulisses Xavier da Silva Neto, Vânia Portela Ditzel Westphalen

**Affiliations:** 1 Graduate Program, Dentistry Course, Life Sciences School, Pontifícia Universidade Católica do Paraná. Street Imaculada Conceição, 1155, Prado Velho 80.215-901, Curitiba, PR Brazil; 2 Core Cell Technology, School of Medicina, Pontifícia Universidade Católica do Paraná. Street Imaculada Conceição, 1155, Prado Velho 80.215-901, Curitiba, PR Brazil; 3 Dental Implant Research Institute of Parana, Rua Manoel Eufrasio, 1000 Juvevê, 80.540-010, Curitiba, PR Brazil

**Keywords:** Entosis, Medical research

## Abstract

This study analyzed the efficacy of autologous platelet-rich fibrin (PRF) in maintaining and recovering cell viability of the periodontal ligament (PDL). The PDL cells were isolated from 45 extracted teeth randomly distributed among 6 groups: 5 min, 1 h, 2 h, PRF 30 min, PRF 1 h and PRF 2 h. In the groups 5 min, 1 h and 2 h (n = 5), the teeth were kept dry in extra-alveolar times of 5 min, 1 h and 2 h respectively. The teeth of the groups PRF 30 min, PRF 1 h and PRF 2 h (n = 10) were kept dry at extra-alveolar times of 30 min, 1 and 2 h followed by immersion in PRF for 45 min. PDL cells were isolated by enzymatic digestion with type II collagenase and dispase, counted and analyzed for viability with Trypan blue vital dye in Neubauer chamber. The variables total number of cells and cell viability demonstrated that in the 5 min, 1 h and 2 h groups there was a decrease after the extra-alveolar dry times of 1 and 2 h. In comparison with the total number of cells, group 1 h, considered immediate reimplantation, did not present statistical difference when compared to the groups PRF 30 min, PRF 1 h and 2 h, a result that demonstrates that PRF assists in cell maintenance and recovery. PRF provided increased cell viability in relation to the different dry extra-alveolar times analyzed (*p* < *0.001*). Autologous PRF presented effectiveness in maintaining and recovering PDL cells from extracted teeth and kept dry for up to 2 h.

## Introduction

Dental avulsion consists of the complete displacement of the tooth from its alveolus, resulting in damage to the cells of the periodontal ligament (PDL) that are attached to the root surface^1^. The most appropriate treatment is the immediate reimplantation^2–4^ and it is not possible to perform it, the alveolar extra time increases and the already damaged PDL cells suffer degeneration^2,5^. In cases of avulsion, the teeth should be stored immediately in a substance that provides nutrition to the cells that remained at its root^3,6^.

The storage media should have optimum pH and osmolarity to maintain PDL cells during extra-alveolar time. In general, the media are not able to replenish the metabolites of PDL cells^7–9^.

Substances such as milk and egg white are means of storage accessible to the population and have chemical and physical properties that contribute to PDL preservation for up to 6 hours^3,10–16^. However, storage in these substances limits the clonogenic and mitogenic capacity of PDL cells^7,12,17–19^.

Hank’s balanced salt solution (HBSS) is the substance that has the highest potential in maintaining the viability of the PDL, since it has pH 7.4 and 280 Osmol/L and contains essential nutrients for the maintenance of cells, being ideal to preserve cells and tissues for the 24-hour period, but is not available^3,12,13,20–22^.

The worst prognosis in cases of avulsion is observed in late reimplants, in the absence of viable PDL cells the root resorptions are expected which lead to early tooth loss. A substance that prevents or delays root resorption of avulsed teeth with dry extra-alveolar time is required.

The use of platelet aggregates, platelet rich plasma (PRP) and platelet rich fibrin (PRF) are described for a variety of dental and medical regenerative procedures, in order to assist in the repair of wounds used as scaffolding, promoting angiogenesis and immunocompatibility^23–31^. Choukroun *et al*.^32^ described platelet-rich fibrin (PRF) that is easily obtained by centrifugation of freshly collected blood and without biochemical treatment, in which there is a predominance of platelets and fibrin in different concentrations, with or without leukocytes^23,33–35^.

The concept of tissue engineering is based on three important pillars: the cells, a biocompatible matrix and bioactive molecules responsible for the morphogenic signals.

The PRF presents advantages over other platelet aggregates such as the preparation in a single step with the production of natural blood products due to the absence of anticoagulants^36^, in addition to resulting in a three-dimensional (3-D) structure that favors the delivery and support of Cell sheets in an area of the tissue, which has been destroyed. This fibrin matrix is able to mimic the extracellular matrix in structural terms, and creates an environment that optimizes cell function and as it contains glycosaminoglycans (heparin and hyaluronic acid) presents a strong affinity with small circulating peptides such as cytokines platelets^27,34,37^.

The processing of the  PRF by centrifugation promotes immediate degranulation of platelets which implies significant release of cytokines, in particular: platelet-derived growth factors (PDGFs) that stimulate the mesenchymal cell line and are essential regulators for the migration and survival of these cells, beta-transforming growth factors (TGF-β1) that induce to collagen I synthesis, fibronectin fibroblasts, osteoblast and the insulin-like growth factors (IGFs) are potent agents of cell protection, because they increase the potential for survival for most cell types^25,38^.

In dentistry, studies with  PRF are more concentrated in the areas of implant dentistry and periodontal and most, demonstrate favorable results in the recovery and repair of soft and hard tissues^26,28,37^. This biomaterial has also been tested as a matrix in the process of regeneration of the periodontium of reimplanted teeth, and describes the PRF as a biocompatible and specific matrix for the delivery of therapeutic sheets that would improve clinical efficacy and would sustain cells in the space between the alveolar bone and cementum^39–41^. During the polymerization of fibrin, which is slow due to the absence of activators, there is the intrinsic incorporation of the cytokines  platelet and glycan chains in the fibrin mashes and the local action of these factors result in survival, migration, proliferation and cell differentiation^25,33,42,43^.

Properties of the growth factors signal the possibility of cell recovery necessary to enable PDL cell proliferation by repopulating the naked surface of the dental root and inhibiting the action of osteoclasts^18,37,43,44^.

The physical and chemical properties of fibrin and the local action of growth factors instigate the possibility of using autologous PRF to maintain the viability of the PDL of teeth exposed to dry extra-alveolar time.

The objective of this study was to evaluate the efficacy of the use of autologous PRF in the maintenance and recovery of PDL cell viability of extracted teeth that were submitted to extra time in the dry eye for 30 min 1 and 2 h.

## Results

A sample of 45 teeth was analyzed, which was divided among the research groups The analyzes performed were in relation to the number of PDL cells obtained after the enzymatic dissociation and their viability (Table 1).

**Table 1:** Descriptive Statistics of the variables analyzed according to Group, Kruskal- Wallis non-parametric test.

Variable	Group	Medium	Standard deviation	P value (Kruskal -Wallis test)
Total number of cells	5 min - 5 minutes	2100000.00	343874,98	0,00017
	1 h - 1 hour dry	280000,009	339455,45	
	2 h - 2 hours dry	0.00	0.00	
	PRF 30 min – 30 mindry + 45 min PRF	1160000.00	1489750.54	
	PRF 1h-1h dry + 45 h PRF	655000.00	333360,00	
	PRF 2 h - 2 h dry + 45 h PRF	735,000.00	589445,13	
Viability (%)	5 min - 5 minutes	100.00	0.00	
	1 h - 1 h dry	93.00	8.92	0.00118
	2 hours - 2 hours dry	0.00	0.00	
	PRF 30 min – 30 mindry + 45 min PRF	99.00	7.26	
	PRF 1 h - 1 hour dry + 45 min PRF	93.50	6.53	
	PRF 2 h - 2 h dry + 45 h PRF	99.50	13.23	

**Figure 1:**
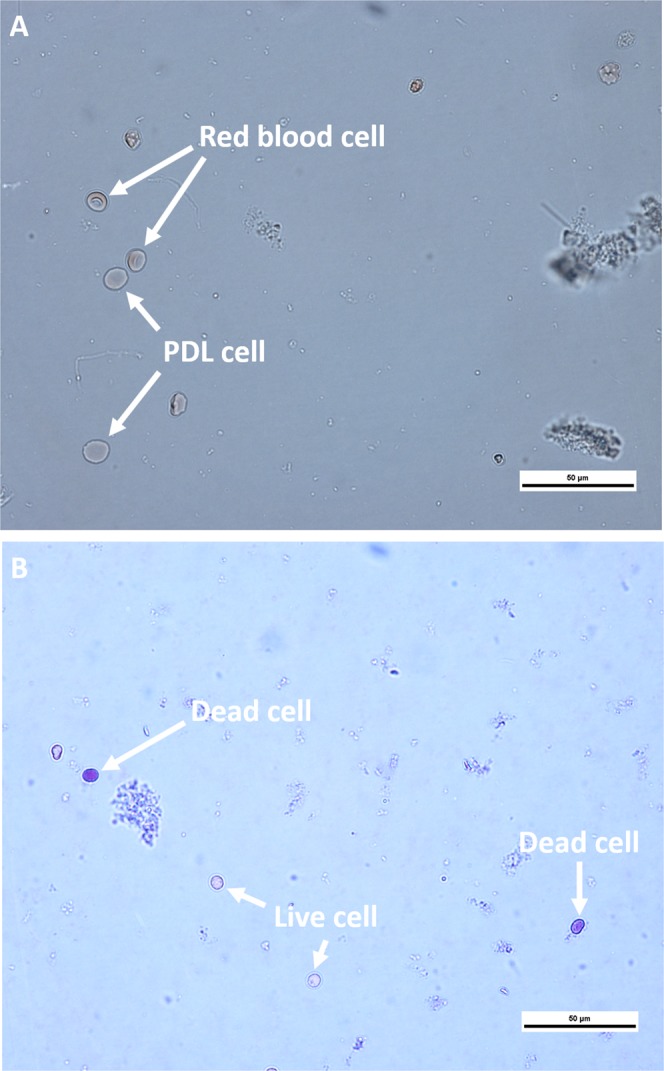
(**A**) Sample photomicrograph after isolation of PDL cells. (**B**) Cell viability analysis.

Cell counting and cell viability analysis were performed by 400x optical microscopy in the samples of all groups, and the mean number of intact and viable cells was determined (Table 1). Cells whose membranes showed irregularities were not counted. Only the whole cells were counted. In the count for the cell viability analysis, the uncolored (translucent) cells were counted as viable and the cells stained in blue were not counted (Fig. 1).

The results of the two variables analyzed demonstrated that in the 5 min, 1 h and 2 h groups, there was a decrease in the time dependent on both cell numbers and viability (*p* < *0.001*).

In the variable percentage of cell viability, we observed a statistically significant difference between group 2 h when compared to groups 5 min and 1 h. This difference was also observed in the groups in which the teeth were immersed in platelet rich fibrin PRF 30 min, PRF 1 h and PRF 2 h (Table 2).

**Table 2:** Comparisons 2 to 2 of cellular viability between groups according to Dunn’s nonparametric multiple comparisons test, applied after the Kruskal-Wallis test to identify statistically significant difference between at least 2 groups (p <0.00118).

Time	Multiple nonparametric comparisons test of six groups	P-value
**1 h** (1 hour dry)	**PRF 2h**-(2 hours dry + 45 minutes PRF)	0,4918
	**PRF 30 min (**30 minutes dry + 45 minutes PRF)	0,4649
	**5 min (**5 minutes dry)	0,0731
**2 h** (2 hours dry)	**PRF 1 h (**1 hour dry + 45 minutes PRF)	0,0142
	**1 h** (1 hour dry)	0,0191
	**PRF 2 h** (2 hours dry + 45 minutes PRF)	0,0007
	**PRF 30 min** (30 minutes dry + 45 minutes PRF)	0,0006
	**5 min** (5 minutes dry)	0
**PRF 30 min** **(**30 min dry + 45 minutes PRF**)**	**5 min** (5 minutes dry)	0,1807
**PRF 1 h** **(**1 hour dry + 45 minutes PRF**)**	**1 h** (1 hour dry)	0,8001
	**PRF 2 h** (2 hours dry + 45 minutes PRF)	0,2493
	**PRF 30 min** (30 minutes dry + 45 minutes PRF)	0,2281
	**5 min** (5 minutes dry)	0,0202
**PRF 2 h** (2 hours dry + 45 minutes PRF)	**PRF 30 min** (30 min dry + 45 min PRF)	0,9576
	**5 min** (5 minutes)	0,167

Dunn’s multiple nonparametric comparisons.

The statistically significant difference between groups 2 h and PRF 2 h confirms that PRF provided an increase in cell viability in relation to the different dry extra-alveolar times analyzed.

## Discussion

The lower the extra alveolar time of the avulsed tooth, the better the chances of success, the half-hour dry alveolar extra time is the gold standard for the reimplantation, until one hour the cells of the periodontal ligament are alive and the prognosis of the immediate reimplantation is adequate, the time of 2 hours was selected, because at that time the cells of the periodontal ligament are already necrotic and the reimplantation performed under these conditions leads to resorption by substitution^4^.

The results of this study showed that the teeth that were stored for 2 h extra-alveolar dry and then placed in the PRF for 45 min did not present significant difference with the groups of 1 h, PRF 30 min and PRF 1 h, in the two variables analyzed, total number of cells and viability. Cell viability analysis by the trypan blue staining method is a widely used and reliable method^45,46^. This result encourages future research *in vivo*, since many teeth that suffer avulsion are prematurely lost after late reimplantation due to the absence of the cells of the periodontal ligament adhered to the root of the tooth. After a dry extra-alveolar period of two hours, the expected adverse conditions can be reduced which would provide a better prognosis, similar to that in the immediate re-implants.

The selected time of up to 2 h dry was used because it is known that in this period the cells of the periodontal ligament are totally extinct^47,48^. This study showed that in this dry alveolar extra time, the percentage of cell viability and the total number of cells was zero. Thus, teeth that would have a poor prognosis could be reimplanted with an adequate condition, since immediate reimplantation prevents the occurrence of resorptive resorption by replacement^4,47^. The dry extra-alveolar time and the quality of the storage medium are responsible for the condition of the PDL at the time of reimplantation and decisive for the favorable prognosis^2,5,46^.

Studies have shown optimize platelet concentrates with remodeling and healing of soft and hard tissues^29–31,34,35,49–51^. During centrifugation, blood is platelet activation and the subsequent release of platelet granules α which are reservoirs of many growth factors and timing of the coagulation cascade activation with thrombin synthesis, responsible for inducing the formation of fibrin. PRF, described as the second generation of these concentrates, has the characteristic of naturally and slowly polymerizing during centrifugation and thrombin concentrations acting on the autologous fibrinogen collected are almost physiological because there is no addition of bovine thrombin or anticoagulants^36^.

Platelet degranulation, among other proteins, involves the release of cytokines called growth factors, capable of stimulating cell migration and proliferation within the fibrin matrix^23,34,35,37,52–55^.

Kobayashi *et al*.^56^ compared the release of growth factors between different preparations of platelet aggregates and PRF preparation protocols at 15 min, 60 min, 24 h, 3 and 10 days, and pointed out that in the preparations of PRF the factors were released gradually over time in addition to containing more growth factors within the fibrin matrix.

This study evaluated for the first timethe periodontal ligament cells viability of extracted teeth in stored dry for 30 min, 1 and 2 h, which were stored for 45 min in PRF, a platelet concentrate of the autologous blood plasma and emphasizes that, only the group of teeth that were kept dry for 1 h did not present a statistically significant difference when compared to groups of teeth that were dry for 30 min, 1 and 2 h, which were immersed in PRF for 45 min. The number of cells obtained in group PRF 2 h may be related to sample variability, since there is no significant difference with group PRF 1 h and PRF 30 min. However, it is important to remember that among the components of PRF there is PDGF-AB that regulates cell proliferation and IL-1β that acts on cell proliferation and apoptosis which may be acting on cells^57^.

The results of this research point to a significant increase in the maintenance and recovery of cell viability and in the total number of cells in the teeth that were immersed 45 min in PRF in comparison to the untreated teeth, where both variables reduced after the dry alveolar extra time of 1 h and after 2 h were constant and equal to zero and corroborate with research that demonstrated that dry alveolar overtime of more than one hour is critical for the survival of PDL cells^1,4,55^.

Hiremath *et al*.^54^ evaluated the viability of periodontal ligament cells using autologous blood plasma in teeth extracted by orthodontic indication and kept dry for 40 min followed by immersion in platelet-poor plasma (PPP) or the combination of (PRF) + (PPP) for 45 min. The combination of PRF + PPP demonstrated a higher number of  live cells and was considered an adequate biological maintenance medium for avulsed teeth. However, this study recommended tests with longer dry extra-alveolar time.

Using the same extra-alveolar time as the 2- hour period Zhao *et al*.^55^ tested the combined use of periodontal ligament stem cells and PRF, only PRF and only PDL cells in the reimplantation of 36 incisors from 6 dogs. The results suggest that the association between PRF and PDL cells can promote periodontal healing and regeneration of PDL tissues of late reimplanted teeth.

Su *et al*.^42^ showed that the release of growth factors present in the PRF membrane is significantly higher in the period up to 60 min, being the ideal time for the application, even though it occurred continuously and slowed in the subsequent 300  min. Based on this data the choice was made to collect blood from the donors 15 min before the end of the dry extra-alveolar time, allowing the centrifugation in an exact time to occur the immersion of the teeth in PRF and thus, to benefit the PDL cells within this period of greater release of growth factors^42,56^.

Studies have shown that the release of growth factors present in the PRF membrane is significantly higher in the period up to 60 min compared to shorter times^42,56^, which led us to apply the PRF during this period of time, even the delivery having occurred continuously, but slower in subsequent times^44^. Based on this data the choice was made to collect blood from the donors 15 min before the end of the dry extra-alveolar time, allowing the centrifugation in an exact time to occur the immersion of the teeth in PRF and thus, to benefit the PDL cells within this period of greater release of growth factors.

In order to isolate PDL cells without damaging them, the enzymatic method described by Pillegi *et al*.^58^. This treatment with collagenase and dispase, allowed maximum cell integrity, demonstrated in the results observed in the group (5 m) 5 min of dry alveolar overtime, in which the variable percentage of viability was equal to 100%. This same result has already been reached in a previous study, which examined the avulsed muscles with 30 min extra dry alveolar time. The teeth were immersed in milk and HBSS and on the method, they concluded that it is effective for assessing cell viability as well, they suggested further investigations with longer periods of dry alveolar overtime^59^.

Saini *et al*.^60^ used this method to evaluate PDL cell viability after immersion in different storage media and stated that the method represents the actual clinical situation because cells are not subjected to long processing times to determine their *status* of viability and point to the advantage of this method, the rapid uptake and cellular integrity.

To quantify the number of viable PDL cells in *in vitro* experiments, a widely used methodology is the Blue Tripan exclusion or color action assay^21,46,61–67^,which makes it possible to evaluate in Neubauer’s chamber the real number of viable cells which are not stained, whereas non - viable cells present is stained in blue due to umentada permeability of the cell membrane^65^. Being considered an effective method for feasibility analysis, we chose this methodology to quantify viable PDL cells.

The survival and recovery of the PDL cells so that they can proliferate and repair the degenerate root surface of avulsed and kept teeth, includes the immersion of these teeth into a substance that can replenish cellular nutrients and guarantee the cells, energy and ions to allow repopulation of the PDL.

The PRF exhibits these properties^46,55^, therefore, it demonstrates strong indications that it may be an effective and available option for the root surface treatment of avulsed teeth that were exposed to extra-alveolar times dry considered critical. However, there is a need for future research on the action of PRF on cell viability compared to synthetic biomaterials as well as other means of preserving the periodontal ligament that may support the conduction of *in vivo* studies, and would further improve our understanding of the effectiveness of PRF in late dental reimplantation.

## Methods

This study was approved under the number CAEE 69663917 of the Research Ethics Committee of the Pontifical Catholic University of Parana and was conducted in accordance with the Consolidated Reporting Standards and the Helsinki Declaration. All participants or their caregivers were informed in a simple and clear signed informed consent about the purpose of the research, prior to the procedure of collection and collection of blood.

The sample consisted of 45 healthy donor teeth that had ages between 13 and 18 years old, of both sexes, with indication of premolar tooth extraction due to orthodontic planning. The teeth should be healthy, vital, with complete rhizogenesis and healthy periodontium.

For the participants under the age of 18 years, a signed informed consent had been obtained from a parent and/or a legal guardian, about the purpose of the research, prior to the procedure of collection and collection of blood.

The 45 teeth of the sample were randomly included in 6 experimental groups that occurred by drawing identical, sealed envelopes with no identification containing the respective group name. The envelope was chosen by the donor participant prior to the exodontia procedures and blood collection for PRF preparation.

### Exodontia

**Figure 2:**
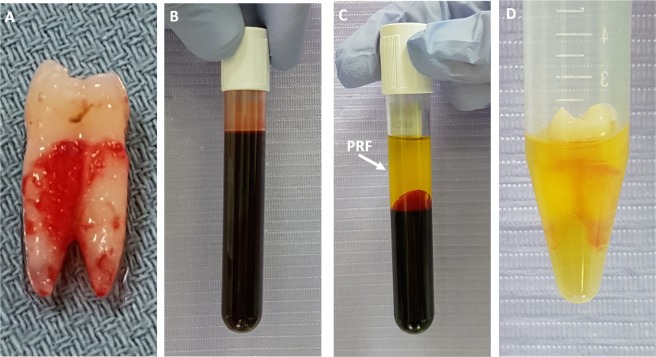
(**A**) Tooth immediately after extraction and washing with PBS. (**B**) Blood was collected by venipuncture. (**C**) PRF after blood centrifugation. (**D**) Tooth  soaking in PRF.

The 45-tooth extraction procedure was performed at the Dentistry Clinic of the Pontifical Catholic University of Paraná (PUCPR) by a single dentist. The patients underwent intra-oral prophylaxis with 2% chlorhexidine mouthwash, followed by local anesthesia with lidocaine 3% with noraepinefrin. Exodontia occurred less traumatically with the use of dental forceps. Immediately after the extractions, the teeth were washed with 0.9% sodium chloride solution and approximately 3 mm PDL from the cervical portion of the roots were scraped with the aid of a No. 15 scalpel blade for discarding the crushed cells during the forceps exodontia. Then, the teeth were packed in Petri dishes and kept dry in contact with the air, being processed later according to the analysis group (Fig. 2A).

### Analysis groups

**Figure 3:**
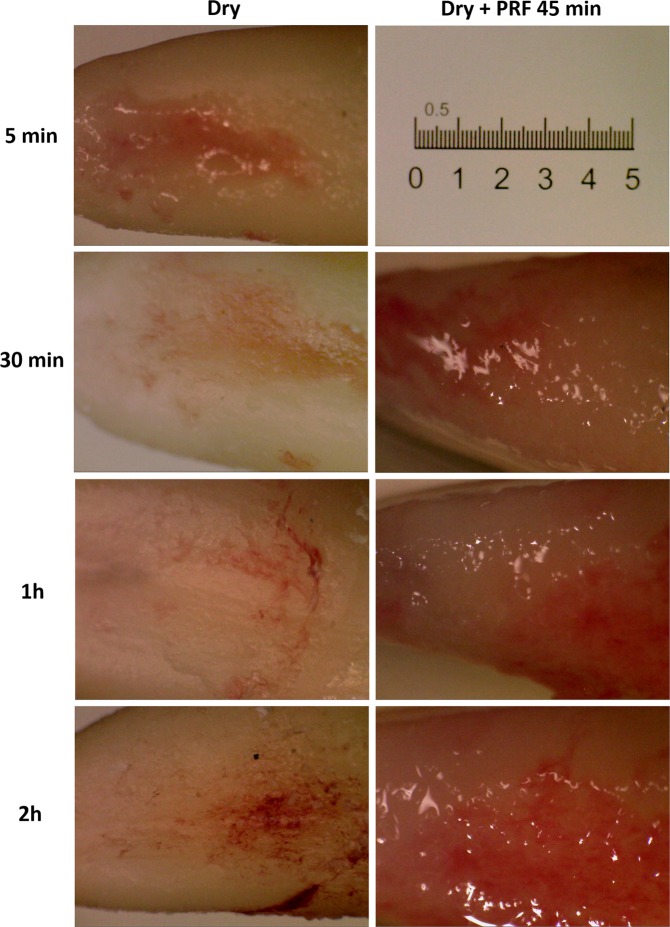
Appearance of dry periodontal ligament at different times (5 min, 30 min, 1 h and 2 h) and after soaking in the PRF for 45 minutes.

The 45 extracted teeth were randomly distributed in 6 groups, nominated of 5 min, 1 h, 2 h, PRF 30 min, PRF 1 h and PRF 2 h (Fig. 3). In the groups 5 min, 1 h and 2 h (n = 5) the teeth were kept dry in extra-alveolar times of 5 min, 1 and 2 h respectively. The choice of the times was according to the worldwide parameters from literature in which, the half-hour (30 min) time is considered the gold standard for the reimplantation, until one hour (1 h) the cells of the periodontal ligament are alive and the prognosis of the immediate reimplantation is adequate and in 2 hours (2 h) time the cells of the periodontal ligament are already necrotic and reimplantation performed under these conditions leads to reabsorption by substitution^4^. In the groups 5 min, 1 h and 2 h (n = 5) the teeth were kept dry in extra- alveolar times of 5 min, 1 and 2 h respectively. The teeth of the groups PRF 30 min, PRF 1 h and PRF 2 h (n = 10) were kept dry in extra-alveolar times of 30 min, 1 and 2 hours, followed by immersion in PRF for 45 min.

### Preparation of PRF

The teeth donors were randomly included in the analysis groups PRF 30 min, PRF 1 h and PRF 2 h, a single blood collection of the median ulnar vein was performed in plastic tubes, white cap and without additives for the PRF preparation. The amount of tubes for the collection of total blood ranged between 1 to 2 tubes of 9 ml, according to the number of teeth extracted from each donor, which varied between 2 or 4 premolars. The platelet-rich fibrin (PRF) was produced using a cervical protocol of 2100 RPM for minutes (RFC - clot = 394 *g*) for 8 minutes using centrifuge Spin plus 5000 - Jiangsu Jinyi Instrument Tecnhology Company Limited - China (45 ° of rotor angulation, with a radius of 80 mm in the blood clot)^68^. The choice of plastic tubes, without additives, had the objective of obtaining an ideal working time, delaying the formation of the fibrin clot.

The collection was performed by a single dental surgeon qualified in venipuncture, according to resolution 158/2015 of the Federal Dental Council, which regulates the use of autologous platelet aggregates for non-transfusion purposes in Dentistry, and occurred in the 15 minutes preceding the dry extra-alveolar time of 30 min, 1 h and 2 h. Immediately after the blood tubes centrifugation, it was collected from the intermediate portion of the plasma (approximately 1 mL) which was dispensed into sterile tubes with a capacity of 15 mL. The teeth were immersed in PRF for 45 min (Fig. 2B–D).

### PDL cell isolation and cell viability analysis

Immediately after the exodontia, blood collection and PRF preparation, the teeth were sent to the Experimental Cell Cultivation Laboratory (LECC) of the Pontifical Catholic University of Paraná to perform the isolation of the PDL cells.

In each of the 45 teeth of the sample the cells were isolated, following the protocol of Pillegi *et al*.^58^, by digestion with the enzymatic solution containing collagenase type II (Invitrogen) (0.2 mg/mL) and dispase (Gibco) (2.4 mg/mL)]. The teeth were immersed in a tube with a capacity of 15 mL containing the enzymatic solution (2.5 mL), remaining under seesaw type agitation at 37 °C for 30 minutes. Then 50 μl of fetal bovine serum (FBS Gibco) was added to the tubes containing the teeth and enzymatic solution for enzymatic inactivation and centrifuged (Centrifuge 5810R - Eppendorf) at 1000 rpm (50.17× *g*) for 5 min. After centrifugation, the teeth were removed from the tubes with a forceps and the supernatant solution discarded. The cell pellet was suspended in phosphate buffered saline (PBS) and the cells were counted in Neubauer’s chamber. For the cellular viability analysis trypan blue dye (Gibco) (0.4%) was used in a ratio of 1: 1. The dead cells show the blue color allowing the analysis of the cellular survival rate.

### Statistical analysis

The results were statistically evaluated by the nonparametric Kruskal Wallis test and Dunn’s multiple nonparametric comparisons test. The level of significance adopted in all tests was 5% in IBM Software (SPSS) Statistics 25.0.

It was concluded that the autologous PRF showed efficacy *ex vivo* in the maintenance and cellular recovery of the PDL of extracted teeth and kept dry for up to 2 hours.

## Data Availability

All data generated or analysed during this study are included in this article.
